# A virtual reality experiment to study pedestrian perception of future street scenarios

**DOI:** 10.1038/s41598-024-55073-x

**Published:** 2024-02-25

**Authors:** Javier Argota Sánchez-Vaquerizo, Carina I. Hausladen, Sachit Mahajan, Marc Matter, Michael Siebenmann, Michael A. B. van Eggermond, Dirk Helbing

**Affiliations:** 1 ETH Zürich, Computational Social Science, 8092 Zurich, Switzerland; 2 California Institute of Technology, Behavioral Economics, Pasadena, CA 91125 USA; 3 University of Applied Sciences Northwestern Switzerland (FHNW), 4132 Muttenz, Switzerland; 4 Complexity Science Hub, 1080 Vienna, Austria

**Keywords:** Engineering, Computational science

## Abstract

The current allocation of street space is based on expected vehicular peak-hour flows. Flexible and adaptive use of this space can respond to changing needs. To evaluate the acceptability of flexible street layouts, several urban environments were designed and implemented in virtual reality. Participants explored these designs in immersive virtual reality in a $2\times 3$ mixed factorial experiment, in which we analysed self-reported, behavioural and physiological responses from participants. Distinct communication strategies were varied between subjects. Participants’ responses reveal a preference for familiar solutions. Unconventional street layouts are less preferred, perceived as unsafe and cause a measurably greater stress response. Furthermore, information provision focusing on comparisons lead participants to focus primarily on the drawbacks, instead of the advantages of novel scenarios. When being able to freely express thoughts and opinions, participants are focused more on the impact of space design on behaviour rather than the objective physical features themselves. Especially, this last finding suggests that it is vital to develop new street scenarios in an inclusive and democratic way: the success of innovating urban spaces depends on how well the vast diversity of citizens’ needs is considered and met.

## Introduction

Rapid urbanization has resulted in the majority of the world’s population living in cities. At the same time, car-centred planning has resulted in the fact that up to one-third of the urban area is allocated to infrastructures for motor vehicles, including roads and parking lots^1^. With increasing population and employment densities within a limited area, cities face numerous challenges, ranging from air and noise pollution over congestion to affordable housing prices and suitable allocation of space^2^.

The trend to provide more of this space to sustainable modes of transport and active mobility has recently resulted in tensions around the allocation and use of road space^3^. At the same time, the growth of cities comes with a growing desire for *public* space. Increasingly, streets are not only viewed as thoroughfares but as places. An allocation of roads to motorized vehicle transport only conflicts with this desire^4^. However, there may be new solutions. The current allocation of space to motorized transport is based on expected peak-hour flows, while the demand for different modes of transport and other uses largely varies with the time-of-day and day-of-week^3^. Therefore, underutilized spaces could be used for other purposes^5^. For example, urban planning concepts such as shared spaces or shared streets integrate vehicular traffic into social spaces by removing typical design elements such as traffic signs and speed humps^6^. As we will see, novel technologies enable even better solutions.

Recently, within the framework of responsive, or adaptable, infrastructures, one explores flexible usage patterns of roads. With the emergence of digitally upgraded infrastructures and autonomous vehicles, new street layouts and scenarios become possible in the near future, which may repurpose spaces currently reserved for cars^7^. These include dynamic reversible lanes^8^, laneless roads^9^, dynamic signalling of right-of-way^10,11^, flex zones and smart curb management^12^ as well as other forms of managed lanes^13^ or curbless flat streets^14^. Our study tests three different street scenarios illustrated in Figs. 5 and 6: *status-quo*, *inLED*, and *curbless*. These distinct implementations (see Table 2) allow one to investigate new street designs that enable adaptive usage paradigms for urban spaces, envisioning a future where there is no static distinction between roadways, sidewalks, and parking lots^15^, but where streets can be used dynamically and flexibly^16^. We would like to emphasize that, here, the term “street” pertains to the entire space located between two buildings and not just limited to the roadways.

So far, there is limited knowledge on how flexible streetscapes should and will be designed, operated, and accepted by people^17^. Among others, it will be essential to study movement patterns of pedestrians in relation to new environments^18^. An understanding of how street design in urban settings affects the behaviour of people can inform the creation of safe urban environments and can support the decision-making of stakeholders when new mobility paradigms arise, including policy-makers, experts, and citizens.

Classical segregated lanes may maximize speeds for motorized vehicles but do not necessarily enhance the overall multi-modal level-of-service. Future streets and vehicle technologies have the potential to overcome this segregation and promote a higher diversity of uses on streets depending on changing needs^17,19^. However, a radical shift in street design may not be accepted by drivers, as they are used to being prioritized. Changes in the allocation of space (e.g. reduction of lanes or parking lots, traffic calming, pedestrianization, expansion of transit and bike lanes) or in pricing schemes (e.g. parking fees, mobility pricing) often face resistance^20^. Therefore, determining the acceptability of measures to be taken and their suitable communication is crucial for successful changes in street design or operation.

For this, it is important to consider the perspectives of all road users and provide them with incentives to embrace the changes in street design and operation, such as a reduction in speed in favour of interoperability and reduced accident severity. *Our study proposes that intrinsic motivation to cooperate can be enhanced by perceived improvements in the quality of mobility.* Implementing changes in traffic flows that promote a slower yet more continuous traffic flow may help to improve the acceptability of speed limits for car drivers. Moreover, such changes may enhance the safety of all road users and create a more equitable and sustainable transportation system based on shared and flexible use of street spaces. Consequently, it may adapt more effectively to changing demands, easing traffic only when needed and promoting space for mobility modes different from private motorized vehicles, thereby reducing undesirable pollution. By prioritizing the needs of all road users and emphasizing the importance of accessibility, comfort, and safety, one may enhance the overall livability of urban areas and foster a more inclusive and sustainable city community.

Current engineering efforts to improve autonomous driving focus, for example, on enhancing sensing capabilities, intervehicle and car-to-infrastructure communication, vehicle safety, and trust in navigation^21–23^. However, the impact of disruptive mobility paradigms on urbanism and traffic, such as adaptive patterns of street usage, is not yet well enough understood. To assess this impact, it is necessary to gather feedback from human subjects, particularly from vulnerable road users. In this paper, we put a particular focus on pedestrians in urban areas, given their high fatality and injury rates^24,25^. Traffic planners will need to better understand how *pedestrians* perceive novel usage technologies and paradigms: are they seen as a safety threat, causing psychological distress, or do they promote a more variable and diverse use of urban spaces? Therefore, we aim to provide new insights that may eventually inform future urban planning and design decisions and ensure that the needs and perspectives of various road users are taken into account.

For this, we use virtual reality (VR), a research tool that has been previously used to assess crossing behaviour^26^, study interaction between pedestrians and autonomous vehicles^27^, communicate future urban designs^28^ and assess the willingness-to-cycle^27^. The advantages are, among others, that it is possible to replicate natural pedestrian behaviour in virtual environments inspired by real-world settings^29^. This allows one to communicate future designs and technology in a realistic, immersive and safe fashion, with relatively low costs and high ecological validity^30^. In fact, a comparison of virtual with real environments reveals that individuals make similar decisions when studying crossing behaviour^31^ and pedestrian behaviour matches real-life norms^32^. That is, VR can help imagine future technologies and their impact better than text-based surveys^33^. In particular, vulnerable users such as pedestrians and cyclists are cognitively and psychologically influenced by VR environments, similarly to the corresponding real-world environments^34^.

Our experiment has three primary goals. First, we aim to test new street scenarios that address the challenges discussed above and promote a more sustainable approach to urban infrastructure design, reducing the negative impact of transportation centred on private vehicles and diversifying and flexibilizing human uses on streets. Second, we aim to investigate how these scenarios are perceived by the public, including their perceived safety, and how they impact travel patterns. Third, we aim to evaluate the foundational elements of communication campaigns that introduce new street scenarios to the public. Specifically, we focus on assessing the effectiveness of video presentations, comparing their impact when delivered in *sequential* as compared to *parallel* formats. Our focus is on examining the perceived safety of various street designs and understanding the psychological responses to these settings, as they significantly influence mobility patterns. By doing so, we aim to facilitate a shift in the prevailing mobility culture, which currently gravitates around motorized vehicles, towards a more eco-friendly and sustainable approach to street usage. Through our research, we hope to provide insights that can inform future urban planning and design decisions and promote the adoption of more inclusive and sustainable transportation policies.

**Figure 1:**
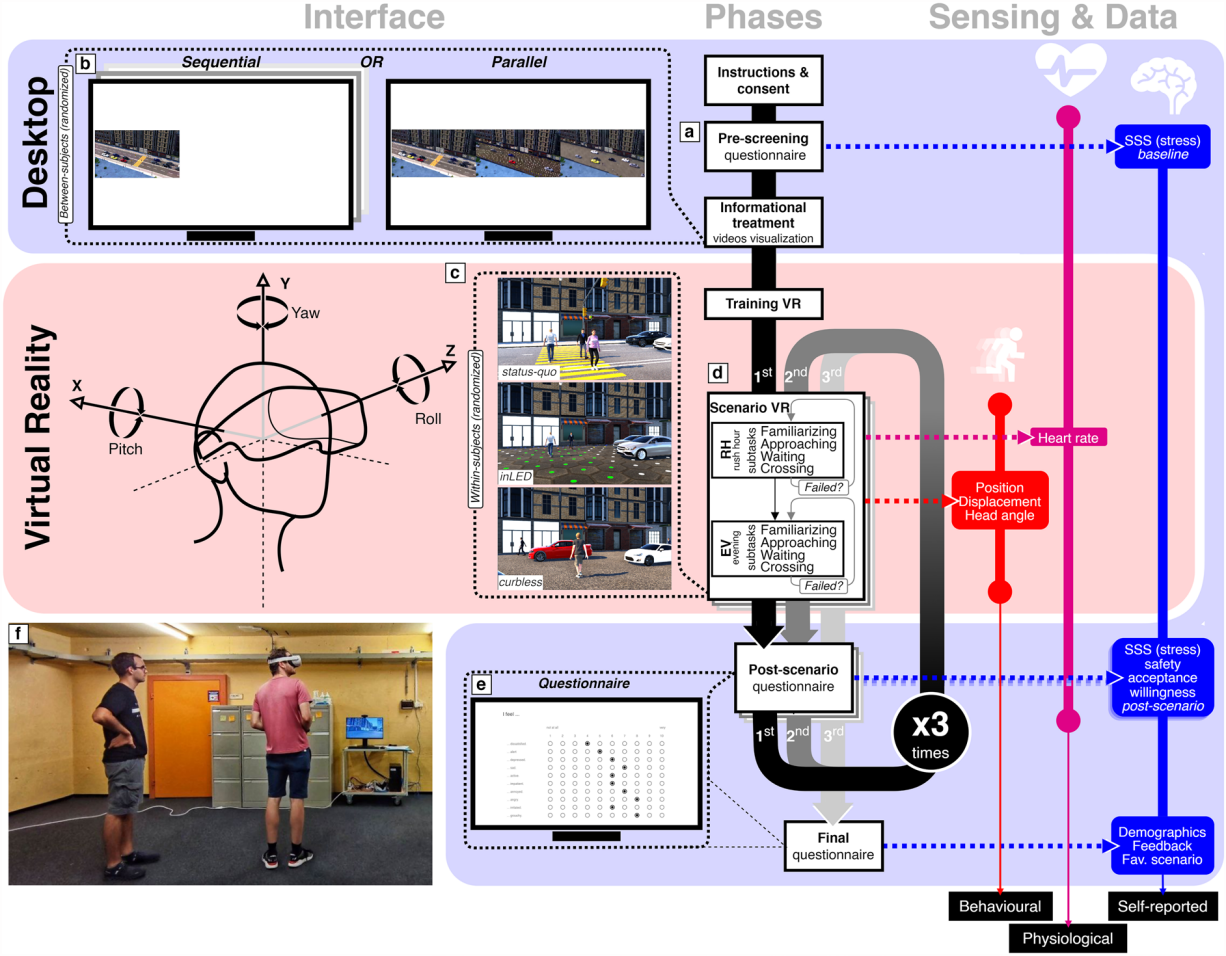
Design, phases, devices and data collection during the experimental setting.

To achieve our goals, we designed and conducted a VR experiment, which focused on the pedestrian perspective (Fig. 1). Specifically, we tested three different street design scenarios that emphasize the concept of shared space and incorporate emerging technologies such as smart pavements and autonomous vehicles, which will become usable in the near future. This was our first goal.

In our VR experiments, participants were asked to cross a virtual street in three different virtual environments (within-subjects factor), each representing a different street design scenario (either *status-quo*, *inLED*, or *curbless*). The order of these scenarios was randomized across participants. Through this, we aimed to quantify user perception and understand the impact of these scenarios on safety and travel patterns. To achieve this, we collected three types of data: self-reported preferences, behavioural data and physiological responses, including heart rate as well as head and body movement patterns. This was our second goal.

Furthermore, to investigate how these changes should be best communicated to the public, we performed a between-subjects treatment. Assigned randomly to each information treatment group, half of the participants watched the new traffic scenarios *sequentially* in an isolated way, while the other half watched the new street paradigms in *parallel* on a split screen. We hypothesized that the split-screen approach supports comparison and helps to recognize the advantages and disadvantages of each scenario better than watching videos sequentially. Ultimately, the insights we gained from this study hope to eventually support the design and implementation of future transportation policies that are more inclusive and sustainable. This was our third goal.

## Results

Our experimental study involved 43 participants with a mean age of 24.76 ($\pm \, 3.73$) years, comprising 62.8% (n = 27) female individuals. The majority of participants were students.

### Number of crossing attempts

Participants were briefed to cross the road safely (i.e. without being hit by a car). Each participant experienced two different configurations (morning rush hour and evening), as illustrated in Fig. 5, in three scenarios (*status-quo*, *inLED*, and *curbless*), as shown in Fig. 6 (for more details see Table 2). Table 1 displays the number of participants by scenario (columns) and treatment (rows). Each participant was given two attempts per configuration in each scenario to cross the road (d in Fig. 1). Depending on the number of failed attempts, this resulted in a somewhat different total number of sessions per scenario and per treatment. Overall, with only $4.5\%$ there was a low rate of failed attempts, but there was a significant impact of the scenario type on the failure rate (n=266, $p_{two-sided\ Fisher}=1.1e-04$). Most of the failures occurred in scenario *inLED*. Apart from this, there were no significant differences in failure rates observed across treatments (*sequential* or *parallel*), indicating that this intervention had no significant impact on performing the task (n=266, $p_{two-sided\ Fisher}=1.0$).

**Table 1:** Summary of participants and recorded sessions.

		Individual participants	Sessions			*Total*
			Scenarios (within-subjects randomized order)			
			*Status-quo*	*inLED*	*Curbless*	
Information treatments (between-subjects randomized groups)	*Sequential*	21	42	47	42	131
	*Parallel*	22	44	48	43	135
	*Total*	43	86	95	85	266

Rows display the number of participants per information treatment group (*sequential*, *parallel*). Columns in the right-most part of the table show the number of sessions per scenario (*status-quo*, *inLED*, and *curbless*).

### Stress, safety, willingness to cross, and attractiveness

Directly after completing the crossing for each scenario, participants responded to a brief structured survey. This survey includes a Short Stress State (SSS)^35^ questionnaire, and questions regarding safety, willingness to cross, and the perception of the attractiveness of a scenario (see “6.6Questionnaires”). to address repeated measures of non-parametric variables, we employed an Aligned Rank Transform (ART) ANOVA test^36^ (see “Materials and methods” for more details). Our analysis was structured across three levels: design scenario (Level 1), information treatment $\times$ scenario (Level 2), and information treatment (Level 3).

**Figure 2:**
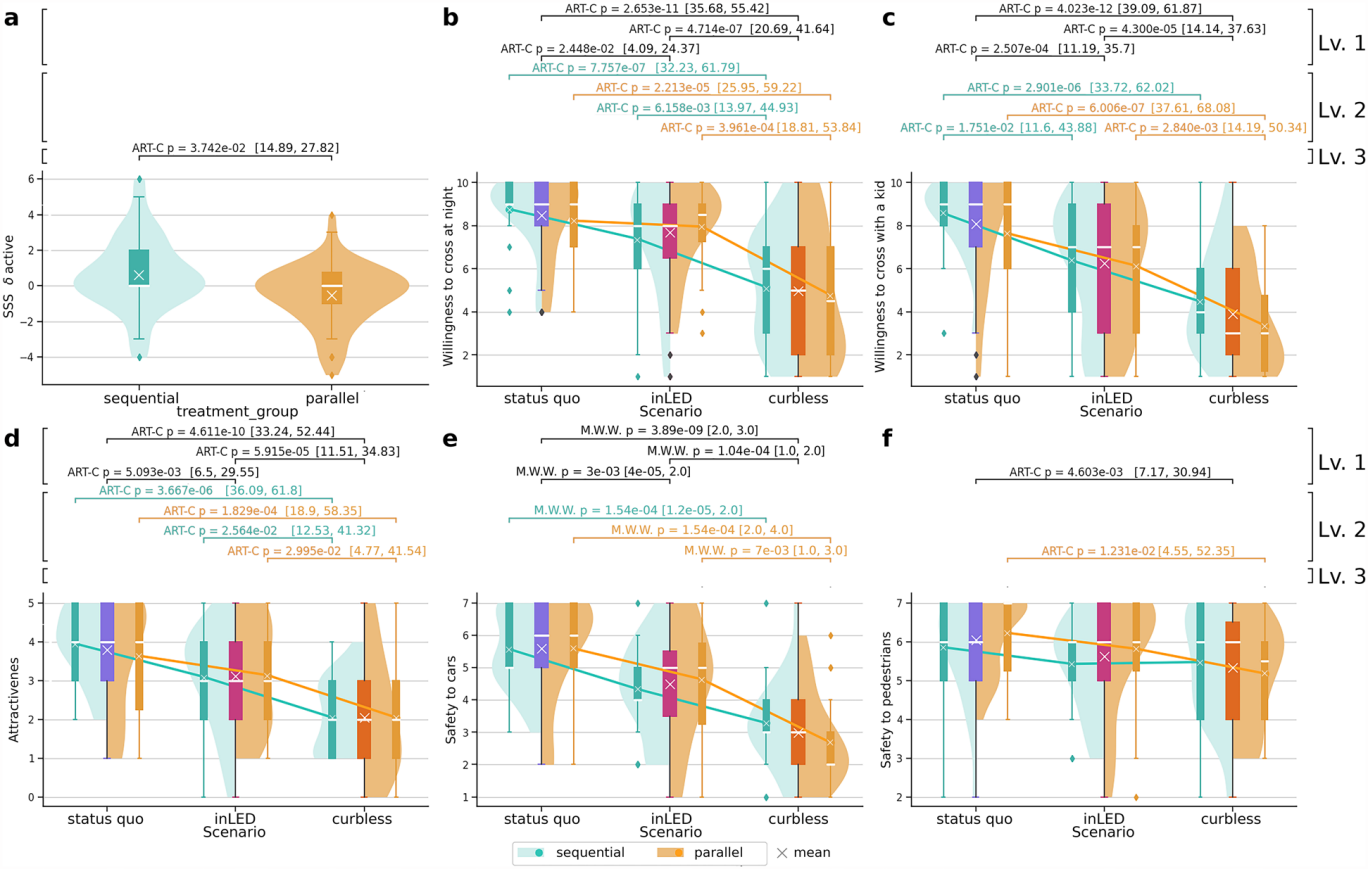
Self-reported responses in the structured survey. Each panel presents a different response to the structured survey, which shows statistically significant differences ($p<0.05$) between *information treatments*, *scenarios*, and their interaction (Information treatment $\times$ Scenario). Confidence intervals for the estimates are shown between brackets. Panel (**a**) displays the distributions for the self-reported “active” feeling on the Short Stress State (SSS) questionnaire. Panels (**b**–**f**) show responses with statistically significant differences for *scenarios* and interactions between interventions. When shown, scenarios are grouped on the *X*-axis, featuring the aggregated distribution as a coloured box plot in blue, magenta, or bright orange. Violin plots show distributions for each information treatment within the same scenario: mint for *sequential* and light orange for *parallel*. They are complemented by corresponding box plots displaying median values and interquartile ranges. For further details on the statistical methods see “Statistical details”.

According to our experiments, scenarios (Level 1) elicit significant variations in responses. Participants’ reactions to the scenarios show the ranking *status-quo* > *inLED* > *curbless* (where “a > b” means that a is more attractive than b). This suggests that participants are less willing to cross unconventional street layouts, which are perceived as less attractive and less safe. This trend is visually apparent through the negative slopes of the lines in panels b–e. In panels e,f, which analyze the differences in the perceived safety for pedestrians and cars (actually, car drivers), differences are observed. Panel e shows a steep slope with significant scenario comparisons (*status-quo* > *inLED* > *curbless*), while panel f exhibits nearly horizontal lines with only one significant comparison (*status-quo* = *inLED* > *curbless*). This suggests that the aversion to unconventional scenarios is caused mainly due to the proximity of cars rather than the proximity to pedestrians.

The analysis of the comparisons of the scenarios across information treatments (Level 2) is facilitated by counting the mint and light orange brackets shown as annotations on top of panels of Fig. 2. We observe that the scenario *status-quo* receives significantly higher ratings than scenario *curbless* in more questions within the *sequential* group (4 questions) compared to the *parallel* group (5 questions). Similarly when comparing scenarios *inLED* and *curbless*: the *sequential* group perceives scenario *curbless* as less likely suited to cross the street at night and less attractive than scenario *inLED*. In addition to these observations, the *parallel* group is additionally less inclined to cross the street with a child and believes the street is less safe regarding interactions with cars.

Even though the small sample size may hinder the ability to draw final conclusions for the within and between-subjects comparisons due to non-significant confidence intervals even in the presence of significant p values, the information treatment (level 3) of our study seems to suggest a likely impact with respect to three additional questions: in scenario *curbless*, participants belonging to the *parallel* group exhibited a reduced willingness to cross the street with a child and appear to perceive the street as less safe regarding the interaction with cars compared to those in the *sequential* group. In essence, the *parallel* group seems to perceive scenario *curbless* more negatively than the *sequential* group. Conversely, in scenario *inLED*, participants in the *parallel* group appear to consider the street to be safer for pedestrians than those in the *sequential* group. In summary, Level 3 differences seem to be most frequently observed in scenario *curbless*, where factors such as willingness to cross the street with a child and perceived car safety are influenced by the presentation of information regarding the scenarios. However, it is needed to further explore these trends with expanded studies (see “Statistical details” and “Limitations and future outlook”).

**Figure 3:**
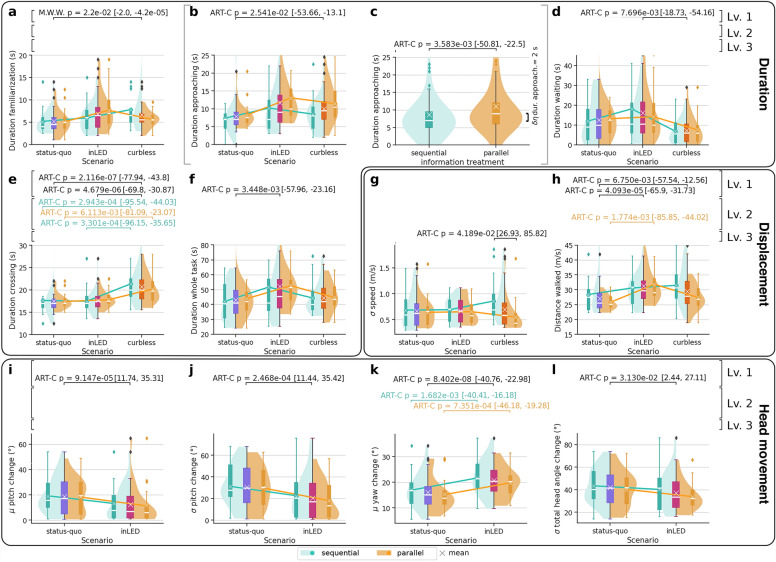
Behavioural responses. Each panel shows a different response measured from the participants’ behaviour, which reflects statistically significant differences ($p<0.05$) between *information treatments*, *scenarios*, or their interaction (Information treatments $\times$ Scenario). Confidence intervals for the estimates are shown between brackets. The participant’s activity was divided into four subtasks, namely *familiarization* with the virtual environment (**a**), *approaching* the crossing (**b**,**c**), *waiting* to cross (**d**), and *crossing* (**e**). The duration in seconds is shown on the y-axis for each subtask in panels (**a**–**e**), and for the whole task in panel (**f**). Panels (**g**,**h**) illustrate differences in uniformity of speed (measured as $\sigma _{speed}$) and the total walked distance within the VR environment. Panels (**i**–**l**) highlight differences in head movement. Specifically, we report the mean ($\mu$) and standard deviation ($\sigma$) of change in pitch (i.e., looking up or down, see panels **i**,**j**) and yaw (i.e., turning head left or right, see panels **k**) as well as the cumulative total angle change in direction of the three axes per second (panel **l**). When shown, scenarios are grouped on the *X*-axis, featuring the aggregated distribution as a coloured box plot in blue, magenta, and bright orange. Violin plots show distributions for each information treatment within the same scenario: mint for *sequential* and orange for *parallel*. They are complemented by corresponding box plots displaying median values and interquartile ranges. For further details on the statistical methods see “Statistical details”.

### Duration

Overall, the largest differences concerning the *duration* of the experiment are found in the subtasks approaching and crossing (panels b,c,e in Fig. 3). The results indicate that participants in the *parallel* treatment took significantly longer to approach the crosswalk compared to those in the *sequential* treatment ($N=266$, $p_{adj\ BY}=3.58e-03$), which is as well suggested by comparison with the *inLED* scenario. Similarly, the duration of waiting (panel d in Fig. 3) was highest in the *inLED* scenario ($p_{adj\ BY}=7.70e-03$). The general trend of increased duration for the *inLED* scenario was also reflected in the duration of the whole experiment (panel f in Fig. 3, $p_{adj\ BY}=3.45e-03$). The average total duration of the task increased from 43 s (± 22 s.d.) in the scenario *status-quo* to 51 s (± 28 s.d.) in the *inLED* scenario, while for *curbless* the average duration of the entire task was 44 s (± 17 s.d.). Overall, the average duration of the whole task for all the scenarios and information treatment is 46 s (± 23 s.d.).

The findings for crossing time (panel e) were in contrast to those for waiting and approaching. Specifically, participants took significantly longer to cross in the *curbless* scenario compared to both the *status-quo* ($p_{adj\ BY}=2.12e-07$) and *inLED* ($p_{adj\ BY}=4.68e-06$) scenarios. This result is consistent with the fact that the *curbless* scenario was the only one without a traffic light or in-pavement LEDs, allowing participants to take as much time as needed to cross. This could potentially explain the longer crossing times observed in panel e.

In comparing treatments across scenarios (level 2), the results indicate that the *sequential* treatment exhibited a strict ordering of *status-quo* < *inLED* < *curbless*. Likewise, the *parallel* treatment showed an ordering of *status-quo* $\approx$ *inLED* < *curbless*.

### Displacement

In the following, we examine the participants’ *movement patterns*, which encompassed both their actual physical movements in a play area of 10 m $\times$ 5 m and their ability to “teleport” within the VR environment. We first calculated the participants’ *speed*, which was defined as meters moved per second. The standard deviation of speed ($\sigma _{\text {speed}}$) served as a proxy for the uniformity in movement pace. According to panel g in Fig. 3, the standard deviation $\sigma _{\text {speed}}$ differed significantly across treatments in the *curbless* scenario. Specifically, participants in the *sequential* treatment exhibited more variation in their movement speed compared to those in the *parallel* treatment. Our analysis of the overall distance walked (panel h in Fig. 3) revealed the following ordering: *status-quo* < *inLED* $\approx$ *curbless*. This effect was particularly pronounced in the *parallel* treatment, as we observed a significant difference in distance walked between the *status-quo* and *inLED* scenarios for this treatment.

### Head movement

During the VR experiment, *head movement and displacement* was logged to quantify the exploration of the environment^37–39^. The head movement for scenario *curbless* was not recorded due to a bug. Consequently, we only report significant differences between scenarios *status-quo* and *inLED*. Our analysis shows that pitch (i.e., looking up and down) and yaw (i.e., turning head left and right) differed significantly across the treatments in terms of both, their mean ($\mu$) and standard deviation ($\sigma$) (panels i–l in  Fig. 3). Specifically, participants looked less up and down in the *inLED* scenario compared to the *status-quo* scenario, while they turned their head more from left to right in the *inLED* scenario compared to the *status-quo* scenario. In other words, the scenario appeared to influence participants’ visual exploration patterns: the *inLED* scenario required participants to focus more on exploring the ground plane, while the *status-quo* scenario needed more exploration between the ground and eye-level plane.

**Figure 4:**
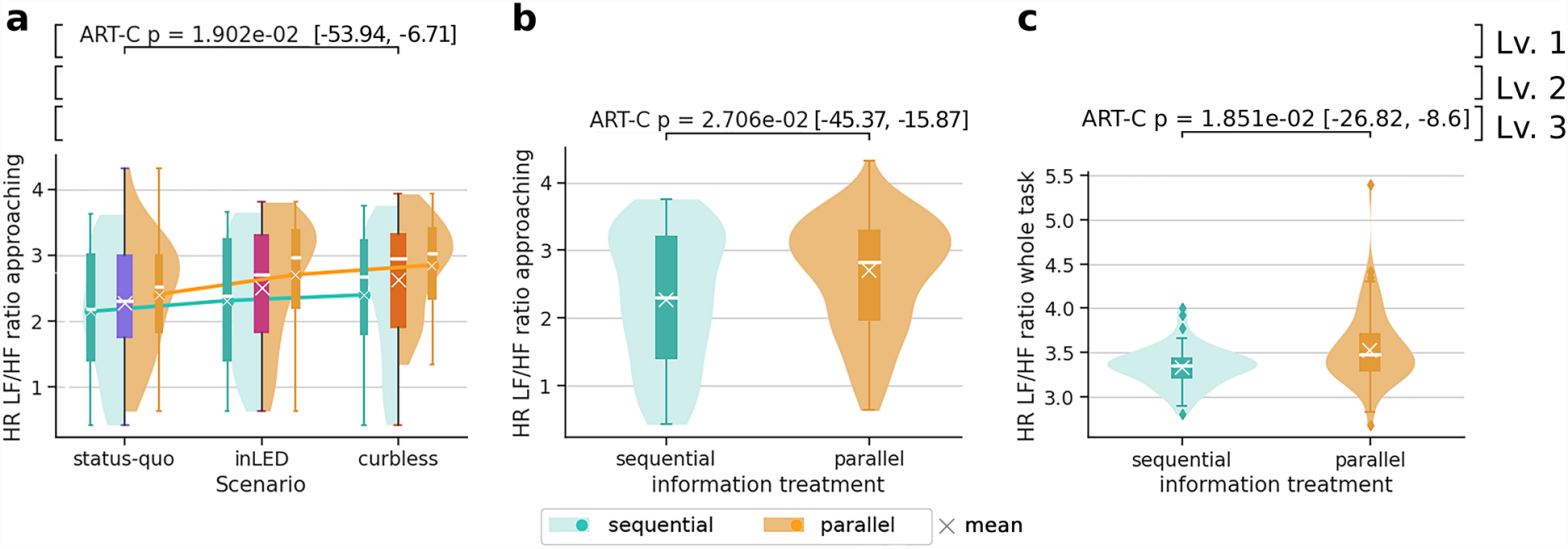
Physiological responses. Each panel presents statistically significant differences ($p<0.05$) of low-frequency/high-frequency (LF/HF) ratio metric based on the heart-rate data for different subtasks within the VR session task: subtask *approaching* in panels (**a**,**b**) and whole task in panel (**c**). Confidence intervals for the estimates are shown between brackets. When shown, scenarios are grouped on the *X*-axis, featuring the aggregated distribution as a coloured box plot in blue, magenta and bright orange. Violin plots show distributions for each information treatment within the same scenario: mint for *sequential* and light orange for *parallel*. They are complemented by corresponding box plots displaying median values and interquartile ranges. For further details on the statistical methods see “Statistical details”.

### Heart rate data

Figure 4, displays the *low frequency* (LF)/ *high frequency* (HF) ratio (see “Heart rate data”). It is interpreted as an index of autonomic balance, where higher values indicate greater sympathetic dominance and lower values indicate greater parasympathetic dominance^40^. Panel a shows a significant difference between the *status-quo* and *curbless* scenarios ($N=266$, $p_{adj\ BY}=1.902e-02$). Moreover, panel c reveals a significant difference in the LF/HF ratio between treatments: *parallel* viewers had a significantly higher ratio than *sequential* viewers ($N=266$, $p_{adj\ BY}=9.925e-03$). Specifically, panel b shows how *parallel* viewers had a significantly higher heart rate when *approaching* the crossing than *sequential* viewers ($N=266$, $p_{adj\ BY}=2.706e-02$).

### Open-answer questions

The analysis of open-answer questions involved a two-stage process. First, all responses were read and labels were generated to summarise the answers in a meaningful way. Second, the labels were assigned using the LightTag software to ensure reproducibility.

The participants provided feedback on the *advantages and disadvantages* of the different street crossing scenarios (see “Questionnaires”). Regarding the *status-quo* scenario, participants commonly noted advantages such as its safety ($n=19$), familiarity ($n=14$), and ease of understanding ($n=10$). However, the most commonly mentioned disadvantage was the wait times of pedestrians and the fact that there was only one designated spot for crossing the street ($n=15$).

The *inLED* scenario received mixed reviews from the participants. Among the advantages, the absence of a need to search for a crossing path for a long time was frequently noted ($n=10$). Moreover, participants appreciated the clear and visible markings of the crossing, particularly at night ($n=1$). Additionally, some participants found the scenario aesthetically pleasing ($n=4$). However, only a minority felt that the scenario was safe ($n=5$). The most commonly mentioned disadvantage was the perceived complexity of the scenario ($n=14$). Participants also found the setting unfamiliar, which could be a potential hurdle to widespread adoption ($n=8$). Interestingly, a considerable number of participants expressed concerns about the expensive cost of implementing and maintaining the inLED scenario ($n=6$).

The *curbless* scenario generated mixed feedback from the participants. Many participants noted the convenience of being able to cross the street whenever they wanted ($n=21$). Some considered the scenario cost-efficient ($n=4$). However, the overwhelming majority of participants did not feel safe when crossing the street ($n=24$). Some participants assumed the absence of visual cues meant that car drivers had no obligation to stop for pedestrians ($n=8$). Furthermore, a few participants found the scenario to be complicated and tiring ($n=6$). Interestingly, some participants also reported that cars frequently stopped too close to them, while they were crossing the street, which was a notable disadvantage ($n=3$).

With regard to the different scenarios, participants were asked for their willingness to change their mode of transportation, and a range of responses was given. The *curbless* scenario received mixed feedback from the participants. Interestingly, the most common response was that participants did not want to be either a pedestrian ($n=8$) or a car driver ($n=8$) in the scenario. This might be due to the perception that scenario *curbless* had an increased probability that cars could hit someone. Moreover, some participants specifically stated a desire to switch from being a pedestrian to being in a car (driver) ($n=6$). However, some participants found being a pedestrian in this scenario to be satisfactory ($n=5$). Regarding the *inLED* scenario, a majority of participants stated that they were comfortable with being a pedestrian in the scenario ($n=7$). Similarly, for the *status-quo* scenario, a comparable number of participants reported being fine with being a pedestrian ($n=8$).

During the study, participants were asked to identify the differences they observed while crossing the street in different scenarios. The majority of participants noted that the crosswalk and traffic lights were the main differences they noticed ($n=24$). Notably, a significant number of participants identified safety as the primary variable that varied across scenarios ($n=12$). Interestingly, some participants believed that the primary difference was in the level of difficulty ($n=4$) or familiarity ($n=4$).

## Discussion

Our primary finding is that unconventional street layouts are less preferred and perceived as less safe in both the structured survey and open-answer questions. These results are mirrored by the heart rate data: we observe greater stress response in the *curbless* scenario, as it is unfamiliar, perceived as dangerous, and requires more cognitive resources to navigate. Hence, in our study, the *status-quo* street design is preferred over unconventional layouts. Nevertheless, in-street technology, such as presented in the *inLED* scenario, is preferred over the *curbless* scenario. Such technology can reserve areas for pedestrians and allows for flexible and adaptive use of space by time-of-day and day-of-week.

The *inLED* and *curbless* are two extreme examples of shared space scenarios. Although there is no clear definition of shared space, all definitions agree that shared space is characterized by the removal of a clear separation between different road users^41^ and integrating vehicular traffic into social space^6^. Research on shared space has resulted in mixed results, varying from pedestrians not minding to share space^42^, to pedestrians preferring dedicated facilities^43^. From a driver’s perspective, shared space results in anxiety and unease to drivers, resulting in possible lower speeds and more attention being paid to other shared space users^44^.

The *open-answer questions* shed more light on the perceived differences between the different scenarios. A frequently mentioned concern was safety, which aligns with the results of the structured question. The frequent mentioning of familiarity by participants suggests the significant cognitive effort required to adapt to new traffic paradigms and a strong preference for the *status-quo* scenario, which could potentially outweigh the benefits of adopting new transportation innovations due to the status quo bias^45^. While the safety distance to stop did not vary between the scenarios from the participants’ perspective, the stopping distance appeared closer for the participants. This insight is valuable for planners, who need to take into account the effects of removing visual cues in street designs.

The *inLED* scenario took the longest to complete, which is consistent with the open-answer questions, where participants mentioned confusion due to the overwhelming information displayed. Interestingly, the *curbless* scenario without a timing mechanism took the longest to cross. This finding suggests that the convenience of crossing the road can be increased by giving more time to pedestrians.

One indirect measure of presence in VR is head movement. Increased head movement indicates that individuals are likely to behave and judge as in reality^46^. In two scenarios, head movement was recorded. Our study found that the scenario influenced participants’ visual exploration patterns, with the *inLED* scenario requiring more focus on the ground plane and participants turning their heads from the left to right (head yaw), and looking more up-and-down (pitch) in the *status-quo* scenario. This is intuitive, as the novel aspect of this scenario was the hexagonal road pavers that needed to be explored, directing more attention to the left and right rather than up and down. Exploratory research furthermore indicated that an increased head yaw, or scanning, might be due to higher anxiety^47^. Lower self-reported safety for the *inLED* scenario also points to higher anxiety in this scenario.

The content analysis of the answers given to the *open-answer questions* delivered further intriguing insights. Participants commented on the diverging maintenance costs for the different scenarios, suggesting that they should be considered not only as road users but also as citizens and taxpayers. This indicates the importance of not only highlighting the immediate benefits and drawbacks for road users but also of considering organizational issues such as costs.

When asked about their willingness to change their mode of transportation in different scenarios, participants provided surprisingly mixed responses. In the *curbless* scenario, many participants were afraid of hitting pedestrians as car drivers and therefore explicitly stated not wanting to drive a car. On the contrary, other participants expressed fears of being hit by a car as pedestrians and, therefore, preferred to be car drivers. These findings highlight the importance of planners to consider the unintended consequences of their designs on the behaviour of road users if the safety and equity of road use are ultimately improved.

When asked to state the differences, many participants identified safety, familiarity, and difficulty as key variables that varied across scenarios. This suggests that participants were more focused on the affordance of the space^48^ and the behavioural effects of the new installations than on physical features. This highlights the importance of considering how citizens use and feel about a space when designing transportation infrastructure.

In our experiments, *parallel* viewers tended to favour conventional scenarios over unconventional ones more frequently than *sequential* viewers. This finding indicates that having the ability to compare three street scenarios enabled participants to focus primarily on the drawbacks (instead of the advantages) of a novel scenario compared to a familiar one. It is likely not coincidental that the presentation of information made the greatest difference in questions relating to the most vulnerable traffic participants, namely children, and the transport mode with the greatest potential to cause harm: a car. Furthermore, our study found that *parallel* viewers needed a longer approach time to cross, implying greater caution than *sequential* viewers. Furthermore, the information treatment appears to influence the comprehension of the scenarios. Specifically, *parallel* viewers showed a more constant movement pace, implying they may have comprehended the single scenario better than *sequential* viewers. These findings are mirrored by heart rate data: *parallel* viewing caused a shift towards sympathetic dominance, frequently associated with stress or alert states.

## Conclusions

### Summary

This study utilized immersive VR to evaluate the feasibility of three distinct street design scenarios that could be implemented in the near future and the impact of communication strategies. The scenarios evaluated were: *status-quo*, *inLED*, and *curbless*. Potential benefits of these layouts were highlighted through a different allocation of street space by time-of-day. Half of the participants viewed three videos depicting the design scenarios sequentially, whereas half of the participants viewed the videos simultaneously in split-screen, prior to being immersed in VR. After performing the task of crossing the street in each of the three scenarios in a randomized order, participants answered a series of questions related to safety and perceived stress, as well as provided open-ended responses. Throughout the experiment, we recorded physiological data (heart rate) and tracked participants’ behaviour through head movements and displacement while immersed in VR.

### Conclusions

#### Value of integrating people’s feedback

Overall, our findings highlight the importance of seeking early feedback in any urban planning action and aligning with current practices of incremental and tactical urban planning. Participants’ assumptions that the absence of visual cues influences car driver behaviour emphasize the importance of street design elements (e.g. markings, signs, delineated areas), to ensure driver awareness of their obligations to stop for pedestrians.

#### Comparing between options improves feedback

We also found that the ability to compare multiple scenarios allowed participants to more easily identify the drawbacks of a novel scenario compared to a familiar one. Physiological data supported our qualitative findings, suggesting increased perceived and measurable stress in unfamiliar scenarios.

#### Communication is important

By comparing two communication strategies, this study points out how this can be as relevant as the actual content of the proposed changes in cities. This highlights the importance of informing people effectively, not only focusing on the technical design of proposals for successful policy-making.

#### Tangible, designed visual cues to support street use are important

Furthermore, we highlight the importance of tangible design elements, such as signs and demarcated areas, which increase perceived safety. We also recommend retaining simple markings as they positively change the perception of space.

#### Consequences and behavioural changes centre people’s attention

Finally, our study shows that participants were more focused on the consequences of changes, the affordance of space, and the behavioural effects of new layouts rather than objective differences. This underscores the need for a user-centred approach when designing streets to meet a community’s preferences and needs.

#### Importance of people-centred urban planning innovation

In conclusion, our study has provided valuable insights into the impact of unconventional street layouts on road users’ behaviours and perceptions. Our findings suggest that such layouts are generally perceived as less attractive, less safe, and less preferred by participants. This highlights the importance of designing streetscapes that meet community needs and preferences and providing opportunities to familiarize oneself with new scenarios. We recommend that planners and policy-makers consider multiple dimensions of street design innovations, including organizational issues, costs, and other benefits and drawbacks for citizens. Additionally, we urge planners to take into account citizen perception to prevent unintended consequences such as the switch to less sustainable solutions.

## Limitations and future outlook

Real-world experiments are, of course, expected to provide the highest ecological validity, but they are unfeasible in the context of the future scenarios studied by us^49^. It is known, however, that the use of VR technologies to test responses of subjects to changes in built environments can provide high ecological and internal validity^50^ through realism^29^ and experimental control^34^. On one hand, ecological validity reflects the correspondence between experimental settings and real-world situations. On the other hand, internal validity allows to isolate causal relationships. Nevertheless, more extensive or longitudinal VR studies would be favourable, as subject familiarity with the scenarios may change over time, thereby increasing the likelihood that novel scenarios would eventually become acceptable. Hence, our VR study might underestimate medium-term public acceptance of new street layouts. Among others, the real-world observation of others navigating safely might enhance perceived safety. However, (perceived) safety issues during a transition phase would probably remain.

Additionally, our focus on futuristic scenarios (reliant on technology that is not yet fully established) may also limit current societal acceptance. In general, head movement and other users’ physiological and behavioural response patterns might be underestimated compared to real-world scenarios, as the VR settings did not feature noise, weather conditions and other sources of environmental variability. Furthermore, a higher diversity of types of street users (including bikes, eScooters, and other vulnerable people) could help to increase the realism of the virtual environment. Accordingly, multi-user experiments might benefit from even higher levels of realism and complexity, in particular regarding enhanced interactions between vehicles, environment and people. Generally, well-controlled VR experiments contrast with real-life unpredictability, which might have a larger impact on physiological responses than our information treatment.

In this context, we want to point out some issues with heart rate (HR) and physiological sensing. While some measurements, for example, those of electrodermal activity (EDA) or heart rates have become an experimental standard to assess stress levels, some would also want to consider respiration rates, pose estimation through computer vision techniques, and eye tracking for gaze and pupil change analysis. Furthermore, the use for example of electrocardiography (ECG), which can improve HR collection data, electroencephalography (EEG), or even functional magnetic resonance imaging (fMRI) scans could provide additional insights. In this context, one needs to consider additional ethical issues, including the question of whether more invasive or pervasive monitoring would be justified by the benefits created. Many of these measurements would not be feasible or desirable in real-world applications or may raise significant issues with regard to the protection of data or subjects. One should also consider the principle limitations of data-driven approaches, particularly when inferred variables are involved, i.e. variables other than those measured or measurable directly. In fact, one should be mindful that many data science applications involve a considerable amount of challenges related to matters of context, interpretation, and causality^51^.

Further note, while the sample size of 43 participants used in this study with repeated measures appears to be limited, it is still comparable to other recent experiments using VR technologies studying pedestrian behaviour in built environments^29,30,34,52–55^. Moreover, our research has still precision and statistical power to provide valuable insights into most of the differences within and between subjects explored. Nevertheless, future research might benefit from increasing the number and diversity of participants. Additionally, the study of effective communication strategies in the context of informing about public policy and urban design would benefit from a more comprehensive and detailed design of alternatives.

Another challenge in VR experiments replicating large-scale environments is space availability to effectively and realistically represent movement through the virtual world. Apart from “teleporting” and controller-based displacement already implemented in this study, the use of treadmills may provide a more consistent collection of data regarding displacement and pose, independently of environment size. Overall, the study of the spatial data could unravel insights regarding user occupancy of space and their relationship with other elements located in the environment.

## Materials and methods

### Experimental design

The experiment was conducted in collaboration with the Decision Science Laboratory at ETH Zurich. The laboratory was responsible for participant recruitment and payment, ensuring that experimental data and personal information were kept separate and safe by design. The study was preregistered^56^ and received IRB approval from the Ethics Commission of ETH Zurich (IRB Approval Date: 2021-01-28, IRB Approval Number: EK 2020-N-183). All experiments were performed in accordance with applicable guidelines and regulations. All participants provided written informed consent before they participated in the experiment and were compensated for their time according to the laboratory’s payment scheme.

**Figure 5:**
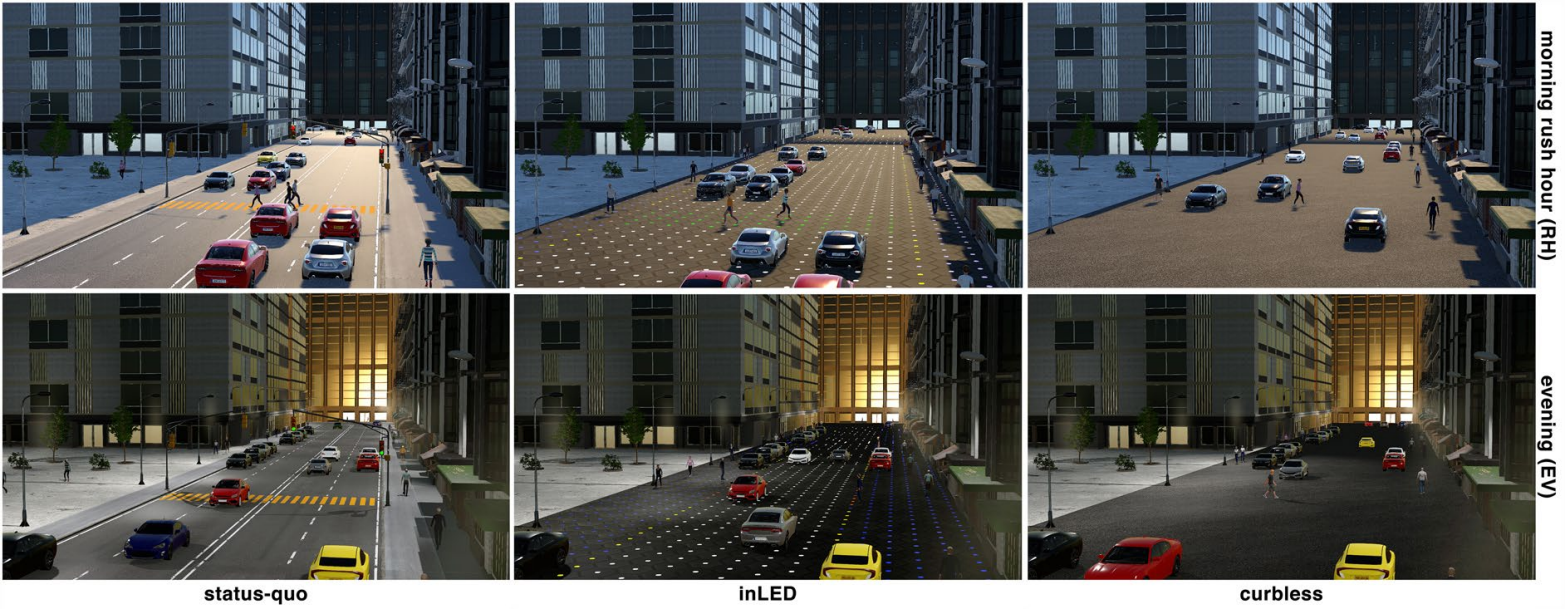
Participants crossed a street wearing VR headsets in three different design scenarios: conventional traffic light controlled pedestrian crossing (*status-quo*, left), dynamic pavement for right-of-way allocation (*inLED*, centre), and flat street (*curbless*, right). Additionally, each traffic scenario was presented for two different times of the day with distinctive lighting, use, and traffic conditions: morning rush hour (top row) and evening traffic (bottom row). For more details, see Table 2.

The participant was briefed to cross the street in three different scenarios. Through this, a participant would experience three different levels and strategies of implementation of new technologies applied to street design with the interplay of autonomous vehicles. To that end, we designed three different street environments (Fig. 5) for our VR experiments: the *status-quo* scenario served as a baseline, with the street characterized by regular sidewalks and a traffic light controlling the pedestrian crossing. The *inLED* scenario allowed for a dynamic allocation of uses, assuming a smart pavement consisting of hexagonal modular pavers^10,11^ incorporating LEDs, which are capable of changing colour based on the intended use of the street. In contrast, the *curbless* scenario relied on smart vehicle sensing and the adaptive behaviour of people, enabling a dynamic use of the space. Consequently, these diverse scenarios put the participants in the position to answer a series of related questions.

**Figure 6:**
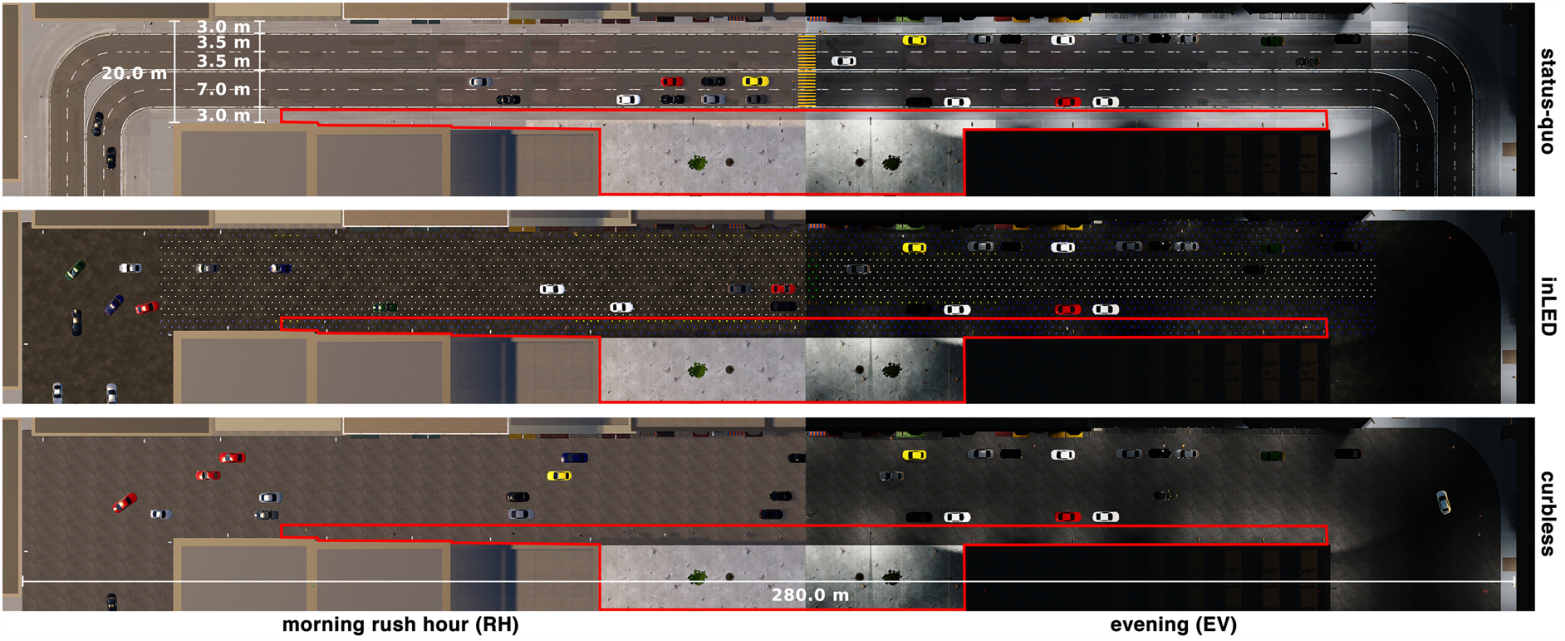
Floor plans of the three designed VR scenarios (*status-quo*, *inLED*, and *curbless*) and the two configurations (*morning rush hour*, and *evening*). The red line highlights the area where teleporting was allowed. For more details, see Table 2.

Apart from the differences in designs, all three scenarios were designed similarly (Fig. 6). Each scenario featured a park adjacent to a straight long road with curves at both ends of the road. The curves blocked the line of sight and allowed for the generation of pedestrians and cars out of sight of the participant. The participant could freely move in this area but was discouraged from moving too far in one direction along one side of the street, namely by limiting teleportation to certain areas (i.e. alongside the street) and potentially by the instructors reminding them that their task was to cross the street.

Each of the three street scenarios was experienced twice in two different configurations by all the participants (i.e., within-subject factor), once during rush hour and once during the evening. The scenarios were designed such that street usage varied throughout the day. During rush hour, the street was characterized by a steady stream of commuters, while in the evening, it was used by families as a space for play and dining. Only the order of the scenarios was randomized across participants.

Prior to starting the VR task, participants filled out an SSS for establishing a stress level baseline for after-treatment differences assessment (see a in Fig. 1). Then, participants watched a 3-min video to become familiar with the traffic environment. The presentation of the videos varied between subjects: participants in the *sequential* group watched videos from a bird’s-eye view for every scenario separately in sequential order matching the randomized order experienced afterwards for performing the task in the three VR scenarios. Instead, participants in the *parallel* group watched the same videos, but on a split-screen, simultaneously in a side-by-side layout, allowing for a direct comparison across scenarios. This gave them additional qualitative information about each street design. In both cases, the window for the video clip of each scenario was depicted in the same size and aspect ratio: i.e., each scenario video window occupied 1/3 of the width of the screen, resulting in *sequential* screen layout having one single video playing with most of the screen empty, and *parallel* visualization having all the three videos filling the entire width of the screen (see b in Fig. 1). In Table 2, a summary of the treatments for the experiment design is shown with a motivation of the interventions, both for the between-subjects information treatments and for the within-subjects scenarios.

The task that participants had to perform was identical in every design scenario and configuration (see d in Fig. 1). Moreover, they had two attempts to perform the task per configuration in case of being hit by a car and failing. To complete the task, participants had three locomotion options, which could be freely switched between (1) physical walking and turning, (2) virtual walking and turning using a controller, and (3) teleportation. Teleportation allowed participants to move automatically to a desired location by pointing the controller and was only possible on the sidewalk and restricted in range to 8 m to prevent participants from simply teleporting across the street (Fig. 6). The virtual walking and teleportation options were introduced due to the large space requirements of the VR setting, although participants also had ample physical space to move around in a 10 m $\times$ 5 m play area. After ensuring a comfortable fit of the head-mounted display, participants were given two controllers and found themselves in a virtual tutorial environment. An instructor guided them through a fixed tutorial sequence for getting accustomed to VR and learning the different locomotion techniques (see f in Fig. 1).

After completing the task, in both configurations for each design scenario, participants were asked to fill in a survey assessing their emotional state and perception of the scenario (see e in Fig. 1 and “Questionnaires”). To complete the survey, they removed the VR headset and switched to a desktop computer. The survey included questions related to stress, perceived safety, and willingness to cross and share the street. At the very end of the experiment, after performing the task in the three scenarios, participants were asked to reply to a set of open-answer questions regarding general impressions and behaviour change based on the scenarios, as well as general socio-demographic data. All these questions that were answered are listed in the paragraph titled ““6.6Questionnaires””. All the questionnaires were designed, hosted and served from the web service provided by Qualtrics and the resulting data stored in their servers.

### Technical details

To gain a deeper understanding of how street design influences the walking experience of pedestrians^57,58^, we captured participants’ physiological responses. Specifically, we measured participants’ heart rate and heart rate variability, as well as their activity levels, using a breast strap (Garmin HRM-Pro) connected through a smartwatch (Garmin Forerunner 55) and a smartphone (HTC Desire 19+) via Bluetooth to the App Garmin Connect to synchronize and store the anonymous data.

For conducting the VR part of the experiment, our setup consisted of a Meta Quest 2 head-mounted display and hand controllers connected to a desktop computer (Intel®Core^TM^ i7-11700 11th generation [8 Cores, 16 MB Cache, 2.5 GHz up to 4.9 GHz], NVIDIA®GeForce®RTX 3070^TM^ 8 GB GDDR6; 32 GB [16 GB x 2, DDR4, 2.933 MHz]; M.2-PCIe-SSD with 1 TB + SATA with 1 TB, 7200 1/min) via Air Link. The VR application was developed in Unity 2020.3.32f1 using the C# programming language. The virtual environment depicted in Fig. 5 was designed with high-quality assets from the Unity Asset Store (“Module Based City Pack” and diverse cars). Realistic, rigged 3D models of pedestrians were taken from Mixamo^59^.

**Table 2:** Characterization of experimental variations: information treatment, scenarios and configurations with its motivations.

	Name	Description	Motivations/aims
Information treatments between-subjects randomized groups (each participant assigned to one group)	*Sequential*	Each video only shows one scenario at a time Each scenario is displayed for 1 min Scenarios are displayed consecutively (one after another) Each scenario is displayed once Size of video window is 1/3 of the treatment *parallel* Total visualization time of all videos is 3 min	Baseline for explanation of scenarios
	*Parallel*	The 3 scenarios are displayed simultaneously Side-by-side window layout Each loop (with the 3 scenarios) lasts 1 min The loop with the 3 scenarios is repeated 3 times Window for each scenario has same size as in *sequential* Total visualization time of videos is 3 min	Enhancing comparison between scenarios Highlighting advantages and disadvantages Synoptic presentation
Scenarios within-subjects randomized order (all participants)	*Status-quo*	Conventional marking and lanes Conventional segregation of roadways and sidewalks Regular crossing with traffic light control	Traffic level of service Safety Maximize max speeds and throughput
	*inLED*	Flat street No curbs, no hard barriers, no hard separations Dynamic pavement lighting to sign changing uses and rights of way	Flexible use of streets Maximizing available space Dynamic setting Minimize speed variations Keep constant speed Matching effective average speed Chasing slower is faster effects Environmental improvement Enhancing IoT and sensing Relies on hard infrastructure
	*Curbless*	Flat street No curbs, no hard barriers, no hard separations No marking lights Conventional pavement Easy to maintain and implement Low infrastructure intervention Low cost Relies on autonomous driving	
Configurations fixed order (all participants)	*Morning * *Rush hour * (RH)	High level of car traffic 2 directions of traffic 2 lanes per direction High level of pedestrian traffic	Increase diversity of urban situations Diversify stimuli and visual noise. Cause more diversity of responses This variation is not randomized, the experiment does not control for this factor
	*Evening* (EV)	Higher level of diversity of uses in the street 2 directions of traffic 1 lane per direction Street parking band combined. Higher level of pedestrian traffic More visual noise Darker environment with artificial light	

### Data collection and pre-processing

Forty-three participants ($n_{participants}=43$) were randomly assigned to the *sequential* or *parallel* information treatment group (between-subjects factor) and completed the task of crossing the virtual street in a randomized order across the three scenarios (within-subjects factor): *status-quo*, *inLED*, and *curbless*. Each scenario was repeated at least twice, following the fixed order for the two configurations: morning rush hour (*RH*) and evening (*EV*) (see panel d in Fig. 1). In cases of task failure, additional attempts were made, providing participants with second opportunities to cross the street again in the formerly failed scenario and configuration (see Tables 1, 2). No participant reported experimental fatigue either verbally during the experiment or in the free-answer questionnaire.

Physiological measurements were taken continuously from the beginning of the experiment until the end resulting in a continuous time series (more details in paragraph “Heart rate data”). Behavioural data were taken separately at every VR session, defined as every run to perform the task in a given scenario and configuration until the participant accomplished crossing the street or failed because of being hit by a car ($n_{sessions}=266$). Behavioural data were computed from the VR environment logs, which recorded the position (*x*, *y*, *z*) and orientation of the VR headset in three angles (*pitch*, *yaw*, *roll*). From this raw log data, *distance walked*, *speed*, and angle change for *pitch*, *yaw*, *roll*, and for*total angle* (as the sum of *pitch* and *yaw*) were computed. The structured self-reported survey (comprising SSS, acceptance, safety perception and assessment of willingness to cross) was answered after completing an entire scenario in both configurations ($n_{structured\ surveys}=129$). Physiological and behavioural time series were synchronized and matched. Later physiological continuous data was cropped according to the starting and ending time of each session to have a combined time series per VR session including physiological and behavioural data.

We recorded the first-person perspective in VR of participants for the entire duration of the experimental session. These recordings were later manually timestamped, which allowed mapping all data points to four different subtasks. The first subtask, which involved participants exploring the virtual environment, was labelled as *familiarization* (1). Following this, participants typically walked towards the curb (or perceived border of the area suitable for cars) in preparation for the road-crossing task, which we referred to as *approaching* (2). Subsequently, each participant had to wait in front of a (virtual) crosswalk, a subtask we termed *waiting* (3) until the red light turned green or he felt it was safe to cross. Finally, participants completed the task by crossing the street, a subtask we called *crossing* (4). The subdivision of the task into these subtasks facilitated comparisons of completion times across the different stages as well as highlighting particular differences within the task. Consequently, time series are cropped as well to match these subtasks. In the case of the lack of one of the subtasks (e.g., because a participant did not wait before starting to walk or before crossing), missing values are imputed with the closest 10 s of data from the contiguous subtasks. Time series for each session are summarised by computing their means ($\nu$), medians ($\eta$), and standard deviations ($\sigma$). These values were later used for statistical analysis.

The answers to the structured self-reported survey were normalized for each participant by computing the differences between each value reported after experiencing a scenario and the initial baseline value registered by each participant at the beginning of the experiment. These differences are later used in the statistical tests.

### Heart rate data

Participants in VR environments are exposed to a variety of stimuli that can elicit a variety of physiological and psychological responses^60^. A variety of sensors, including heart rate sensors, head movement sensors, and displacement sensors, can be used to measure these responses. Heart rate variability (HRV) is a measurement of the time difference between successive heartbeats. It is a useful indicator of the body’s physiological response to stress and other stimuli. Heart rate sensors can be used in VR experiments to track participants’ HRV as they navigate virtual environments. This data can reveal how participants react to various aspects of the environment, such as visual cues or sound effects.

We collected heart rate data continuously, at a rate of 1 Hz from the start to the end of the VR experiment. Outliers in the dataset were removed to guarantee data quality. In such an experimental setting, it is possible to have outliers in the collected heart rate data. Outliers can be caused by a variety of factors, including measurement errors, physiological abnormalities, and participant noncompliance. While pre-processing the data, we looked at the outliers and removed them in a way that did not skew the findings. We resampled and adjusted the data to one sample per second^61–64^ in order to provide a good accuracy to the HRV analysis and sufficient sampling points^65^. Timestamps between different sensing sources, including the VR environment logs and sensing devices, were matched to accurately align data across sources. The whole heart rate time series for each participant was split into individual sessions corresponding to each VR scenario.

In VR experiments, analyzing the power spectral density (PSD) of physiological signals such as heart rate can provide insights into the autonomic nervous system response, which can help in understanding the participants’ emotional or cognitive states^66,67^. The PSD represents the power of various frequencies in a signal mathematically. The PSD gives information about the distribution of power at different frequencies in the heart rate signal in the case of heart rate variability. The PSD’s HF component is linked to parasympathetic (vagal) activity, whereas the PSD’s LF component is linked to sympathetic and parasympathetic activity^68^.

We analyzed the PSD of heart rate data from various participants to investigate the effects of diverse scenarios in a VR experiment on the autonomic nervous system. We used a wearable device (Garmin HRM-Pro) to collect heart rate data, and each participant went through a series of VR scenarios, each intended to elicit a particular emotional response. We used the Welch method to analyze PSD because it reduces noise and gives a more accurate estimate of the PSD^69^. To obtain a more accurate estimate of the PSD, the Welch technique divides the signal into overlapping segments and then averages the periodograms of these segments. The technique reduces noise caused by the signal’s non-stationarity by using overlapping segments, and it improves the estimate of the PSD by averaging the periodograms. The PSD was then integrated into the LF (0.04–0.15 Hz) and HF (0.15–0.4 Hz) bands to determine the total power in each band. The LF/HF ratio was determined as the ratio of LF power to HF power. The LF/HF ratio is a commonly used metric for autonomic nervous system activity. A higher LF/HF ratio is commonly read as a sign of sympathetic dominance or a shift in the balance of sympathetic and parasympathetic activity toward sympathetic dominance. This could be due to stress, nervousness, or other conditions that cause sympathetic activation. A lower LF/HF ratio, on the other hand, is frequently linked with parasympathetic dominance or a shift towards parasympathetic activity, which may be associated with relaxation and recovery^40^.

### Statistical details

To characterise the independent and dependent variables of the experiment, we employed the Shapiro–Wilk test^70^ to check normality and the Levene test^71^ for equal variance. Different statistical methods were applied depending on the type and distribution of the data for assessing the association between variables and the informational treatments and scenarios. However, as the majority of the experiment variables did not follow a normal distribution, we utilized non-parametric tests to evaluate differences across treatments and scenarios.

For categorical variables, both independent (i.e., gender, experience with VR experiments, ownership of a car, driving license, and walking habits,) and dependent (i.e., counts of failed attempts for crossing the street, favourite scenario reported at the end of each experiment) a two-tailed Fisher’s exact test^72^ was used on the contingency table of occurrences for each treatment group due to the small sample size of some of them (i.e., below 5).

We statistically tested the independence of the treatments. The only variables from the socio-demographic data that were not completely randomized and showed unbalance with regards to the treatment groups were ownership of a car ($N=43$ $p_{two\text {-}sided\ Fisher}=1e-03$), driving license ownership ($N=43$ $p_{two\text {-}sided\ Fisher}=2.499e-02$), and reported previous experience in VR experiments ($N=43$, $p_{two\text {-}sided\ Fisher}=5e-04$). Hence, we controlled for the effect of these covariates in combination with the main interventions (*information treatment*, and *scenario*) in all the statistical analyses and they didn’t show any significant effect on the responses ($p>0.05$).

For the analysis of the effects of the interventions, we performed an Aligned Rank Transform (ART) ANOVA test. This allowed us to perform factorial non-parametric analyses when violations in the assumptions of normality and variance were present and it could handle repeated measures^36^ as in the case of the mixed experiment designed.

We tested for the separated effect of each intervention ($Response \sim Information\ treatment + (1|Participant)$ and $Response \sim Scenario + (1|Participant)$) and the interaction between them ($Response \sim Information\ treatment \times Scenario + (1|Participant)$). Given the repeated measures design of the experiment, the main interventions (*information treatment* and *scenario*) were considered as fixed effects, and the repeated measures on the participants on consecutive sessions across scenarios were included as a random effect ((1|*Participant*)).

In those response variables where the random effect for each participant was not significant, an alternative non-parametric Kruskal–Wallis H test for the analysis of variance^73^ was performed for robustness, whose results are reported and shown in figures. No differences regarding the significance of the main effects were found between both procedures.

The sample size (n) for the omnibus analysis of variance changed depending on the response variables and the number of repeated measures taken over the number of participants ($n_{participants}=43$). Dependent variables resulting from the structured self-reported survey were recorded every time after each scenario ($n_{structured\ surveys}=129$, $n_{structured\ surveys\ per\ scenario}=43$, $n_{structured\ surveys\ Sequential}=63$, $n_{structured\ surveys\ Parallel}=66$). Differently, behavioural and physiological responses were taken during each session ($n_{sessions}=266$).

In the case of statistical significance ($p<0.05$) of the omnibus test for variance, it follows a posthoc test to determine the statistical significance of their differences. In the case of using the ART procedure, we performed a contrast multifactor test at three different levels (between *information treatments*, *scenarios*, or their interaction *Information treatments* $\times$ *Scenario*) using the algorithm ART-C, which is an extension of the former to reduce the Type I error rated with higher statistical power than the t-test, Mann–Whitney U test, Mann–Whitney–Wilcoxon test, and ART test, particularly for nonconforming data^74^. Alternatively, for the posthoc test after the Kruskal–Wallis test, we used a two-sided Mann–Whitney–Wilcoxon test^75^. Finally, p values are adjusted for the multiple comparisons using the Benjamini–Yekutieli (BY) method^76^. This adjustment controls Type I error of multiple comparisons based on the *false discovery rate* (FDR), which improves the statistical power when a large number of comparisons are simultaneously tested^77^. BY is a conservative test and compromises slightly the discovery rate by not making assumptions on the dependency of hypotheses. The annotations^78^ on top of each panel in Figs. 2,  3 and  4 display the significant results ($p<0.05$) of the contrast multifactor pairwise posthoc tests for the three levels.

In some cases, after performing a detailed assessment of the distribution of response variables in *information treatment* groups within *scenarios* (i.e., interactions $Information treatment \times Scenario$), and for the within-scenario pairwise contrast tests, a one-sided Mann–Whitney–Wilcoxon test can be used, with a significance level $p < 0.1$, for its higher power due to the particular characteristics of the compared distributions which can allow making a strong hypothesis about the values^79,80^. Simultaneously, to ensure the robustness of the results and control Type II error and lack of precision for the estimates due to small sampling rate^81^, we compute the confidence intervals using bootstrapping^82^ and specific implementations for non-parametric tests^83^. Hence, only differences with significant p values ($p_{ART\text {-}C\ adj\ BY}<0.05$ or $p_{MMW\ adj\ BY}<0.1$) and significant confidence intervals are highlighted in Figs. 2, 3 and 4.

### Questionnaires

Answers to the following questions were recorded using Qualtrics.

#### Open-answer questionnaire

Participants were provided with an open text field, where they could write as much as they wanted in response to the following questions:

- In your opinion, what are the main advantages of each of the three scenarios? What are the main disadvantages?
- What are the most noticeable differences between the three scenarios for you?
- For each or any scenario, would you want to change your usual mode of transportation and why?

#### Socio-demographic questionnaire

The socio-demographic questionnaire was answered at the end of the experiment.

- In which country/city do you live? (dropdown menu w/list of countries)
- In which city were you raised? (dropdown menu w/list of cities)
- How do you define your neighborhood? (city center/downtown, compact walkable neighborhood, low density suburb, rural area/village)
- What is your year of birth? (open field, only NATURAL numbers allowed)
- I identify as (male, female, non-binary/third gender, prefer not to say)
- What is your subject of study/profession? (dropdown menu with ETH-specific choices)
- How often did you take part in experiments? (0, 1–3, > 4)
- Do you have experience with VR? (Y/N)
- Do you have a driving license? (Y/N)
- Do you own a car? (Y/N)
- How many kilometers do you drive per week on average (0, < 10, 10–50, 50–100, > 100).
- What is your preferred mode of transportation for your daily chores (work, groceries, etc.)? (walking, bike, public transportation, motorbike, car)
- Do you walk (> 500 m) daily? (Y/N)

#### Short Stress State (SSS) Questionnaire

All of the items of the short stress state questionnaire^35^ are answered on a 10-point scale, once before and subsequently after each scenario.

- I feel dissatisfied.
- I feel alert.
- I feel depressed.
- I feel sad.
- I feel active.
- I feel impatient.
- I feel annoyed.
- I feel angry.
- I feel irritated.
- I feel grouchy.

#### Safety, willingness, attractiveness

In addition to the short stress state questionnaire, the following questions were asked after participants encountered each street scenario:

- How safe do you feel due to the proximity and number of pedestrians/cars? (1–7)
- How willing are you to cross the street with a kid/at night? (1–7)
- Please rate how attractive you found the scenario as a pedestrian (0–5)

## Data Availability

To ensure the transparency and reproducibility of our research, we will make raw and processed data, analysis scripts and code publicly available after acceptance via an open-access GitHub repository. The working repository, https://github.com/ethz-coss/VR_future_streets, can be accessed upon invitation.
